# Signals from the edges: The cortical hem and antihem in telencephalic development

**DOI:** 10.1016/j.semcdb.2009.04.001

**Published:** 2009-08

**Authors:** Lakshmi Subramanian, Ryan Remedios, Ashwin Shetty, Shubha Tole

**Affiliations:** a Department of Biological Sciences, Tata Institute of Fundamental Research, Mumbai 400005, India; b Department of Biology, Stanford University, USA

**Keywords:** Patterning, Induction, Hippocampal organizer, Hem, Antihem

## Abstract

The early cortical primordium develops from a sheet of neuroepithelium that is flanked by distinct signaling centers. Of these, the hem and the antihem are positioned as longitudinal stripes, running rostro-caudally along the medial and lateral faces, respectively, of each telencepahlic hemisphere. In this review we examine the similarities and differences in how these two signaling centers arise, their roles in patterning adjacent tissues, and the cells and structures they contribute to. Since both the hem and the antihem have been identified across many vertebrate phyla, they appear to be part of an evolutionary conserved set of mechanisms that play fundamental roles in forebrain development.

## 1 Defining the hem and antihem: position, molecular expression domains and signaling molecules

**Fig. 1:**
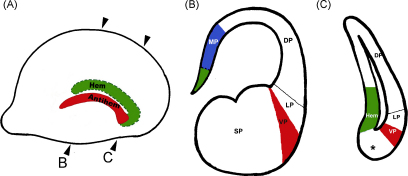
The positions of the hem and the antihem in the dorsal telencephalon. (A) A schematic of an E12.5 telencephalic hemisphere viewed from the lateral face, showing the antihem (red). The hem is schematized on the medial face (green). Rostral is to the left. (B) and (C) are schematics representing mid-level and caudal sections of such a hemisphere, showing the medial pallium (MP) which includes the hem (green) and the hippocampal primordium (blue), the dorsal pallium (DP), lateral pallium (LP), ventral pallium in red (VP/antihem), and subpallium (SP). Asterisk denotes DP tissue present between the hem and antihem at extreme caudal levels, which is the source of the amygdaloid nucleus nLOT2. Modified from Remedios et al. [2] [www.nature.com/neuro/index.html]. (For interpretation of the references to color in this figure legend, the reader is referred to the web version of the article.)

Neuroepithelium that gives rise to the cerebral cortex is flanked by the hem medially, and the antihem laterally ([1,5]; Fig. 1A). Therefore, these two structures are separated by the expanse cortical neuroepithelium (Fig. 1B) except at the extreme caudal pole of the telencephalon, where they almost meet [5], separated by a small domain of cortical neuroepithelium ([2]; Fig. 1C).

Along the medio-lateral axis, the telencephalic neuroepithelium can be divided into four different types of pallial tissue based on gene expression patterns (Fig. 1). The medial pallium (MP) contains the hem and the hippocampal primordium; the dorsal pallium (DP) corresponds to the neocortical primordium; the lateral pallium (LP) is thought to give rise to the piriform cortex; and the ventral pallium (VP), which together with the LP, contributes to specific components of the claustroamygdaloid complex [3,4]. The ventricular zone of the VP is identified as the antihem [5]. An adjacent subpallial region, the dLGE, is also thought to contribute to the amygdaloid complex ([4,6]). The VP and the dLGE lie on either side of the pallial–subpallial boundary (PSB; [7]). A prominent pallisade of radial glial fibers delineates the PSB, originating in a region of the ventricular zone termed the “corticostriatal junction” [8], and extending up to the pial surface in the region of the piriform cortex and amygdala.

*Dbx1*, a transcription factor, is restricted to the VP ventricular zone [4,7,9]. This exclusive expression of *Dbx1*, as well as an enriched expression of the secreted frizzled related gene *sFrp2,* serves to delineate the antihem from adjacent domains [1,5,7,10]. The VP and the adjacent dLGE both share an enriched expression of *Pax6*. In the VP, this expression is limited to the ventricular zone (the antihem), whereas in the dLGE *Pax6* expression extends into the mantle [7].

The hem and the antihem were proposed to be important embryonic signaling centers on the basis of their locations, flanking the cortical neuroepithelium, and their enriched expression of several different types of signaling molecules. The hem expresses signaling molecules of the *Wnt* family, Wnt2b, 3a, and 5a. Several members of the *Bmp* gene family are expressed in broader domains that include the adjacent choroid plexus and/or hippocampal primordium [11,12]. The antihem expresses epidermal growth factor (EGF) family members, a fibroblast growth factor *Fgf7*, as well as a *Wnt* signaling inhibitor *Sfrp2* [1,5]. Of the several EGF family members, ligands *Tgf*α, *Nrg1* and *Nrg3* are concentrated at the antihem. *Egf* is itself expressed throughout the ventral neuroepithelium, but is not concentrated at the PSB [5,13]. *Sfrp2* is intensely expressed in the antihem, and more weakly in the rest of the telencephalic neuroepithelium [1,5]. Members of this family bind directly to Wnts and act as Wnt antagonists [14–16].

In the subsequent sections, we will review the mechanisms that regulate the positions of the hem and the antihem, and how these positions enable the signaling centers to control the structural organization of different brain structures.

## 2 Specification of the hem and the antihem

**Fig. 2:**
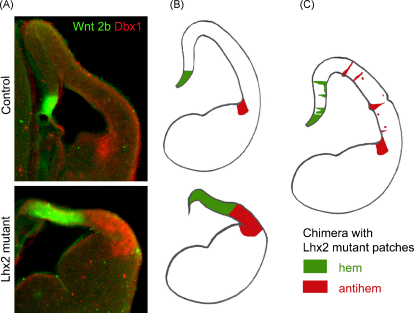
Lhx2 suppresses hem and antihem fate in a position-dependent manner. (A) Hem marker Wnt2b (green) and antihem marker Dbx1 (red) reveal these structures separated by the cortical neuroepithelium in a control E12.5 brain. In the Lhx2 mutant both the hem and the antihem are expanded and there is no intervening cortical neuroepithelium. (B) Schematics representing these data. (C) In a chimeric brain with Lhx2 null clusters scattered amidst wild-type neuroepithelium, the medial null patches take on hem identity whereas lateral patches differentiate into antihem. From Mangale et al. [17] [www.sciencemag.org]. (For interpretation of the references to color in this figure legend, the reader is referred to the web version of the article.)

Along the rostro-caudal axis, the antihem is more pronounced rostrally, appearing at levels anterior to the hem. In contrast, the hem is seen from mid-levels, and is most prominent caudally, persisting at levels where the antihem is no longer present [5,12,17]. These positions parallel the graded expression of developmental control molecules in the telencephalon: *Pax6* is expressed in a rostrolateral (high) to caudo-medial (low) gradient whereas *Lhx2* and *Emx2* show the opposite gradients [18,19]. Pax6 is required for the specification of the antihem ([1,5]). Lhx2 suppresses both hem and antihem fates, and both structures are expanded in the *Lhx2* mutant [17,20,21]. The hem and the antihem are non-cortical in that they do not contribute to the hippocampus or the neocortex. However, cells of the prospective cortical primordium take on either hem or antihem fate in the absence of Lhx2, revealing a fundamental commonality between these two fates. Studies using embryonic stem cell chimeras have demonstrated that *Lhx2* null cells become hem if located medially, and antihem if located laterally ([17]; Fig. 2). It is unknown how this positional control of hem versus antihem fate choice is regulated. Attractive candidates are early-expressing transcription factors that are themselves graded in expression, such as *Pax6* and *Emx2* [22], or *Foxg1*, which suppresses hem fate, and appears to be required for lateral fates including that of the antihem [23,24]. In the dorsal telencephalon of the *Foxg1* mutant, medial fates are expanded and lateral fates are missing [24]. In mosaic embryos created by tamoxifen-induced gene disruption of *Lhx2*, medially located *Lhx2* null patches do not express *Pax6* or *Foxg1*, whereas laterally located *Lhx2* null patches express both these genes [17]. While this is entirely consistent with the requirement of *Pax6* and *Foxg1* for antihem fate, it still does not explain how these differences between medial and lateral Lhx2 null patches is brought about in the first place. This remains a fundamental open question: to understand the early patterning of the telencephalon into distinct signaling centers flanking a territory of “responding” tissue, the cortical neuroepithelium.

## 3 Molecular mechanisms that act to position and specify the cortical hem

Several molecular models have been proposed to explain the position of the cortical hem in the telencephalon. These studies seek to explain the mechanisms which define the rostro-caudal as well as the medio-lateral boundaries of this signaling center. *Fgfs* expressed by the anterior neural ridge (ANR) are fundamental regulators of mid-line patterning [25]. They activate midline expressing transcription factors and repress *Lhx2* and help establish the mid-line domain within the telencephalon prior to invagination. At the same time, Bmps from the roof plate restrict the extent of the *Fgf* expression and are themselves repressed by the Fgfs [26–28]. Fgfs also repress *Wnt* genes whose expression defines the cortical hem [27]. This cross regulation between two groups of secreted signals helps to define a caudo-medial position for the hem.

This domain is further refined by cross-regulatory interactions between transcription factors *Emx2* and *Pax6* [29]. In particular, Emx2 appears to specify a caudo-medial domain in the telencephalon which contains the cortical hem as well as the hippocampus. Emx2 may act by restricting the anterior region of *Fgf* gene expression [27]. Furthermore, Emx2 functions as an effector of the canonical Wnt signaling from the hem to regulate proliferation within the caudo-medial region [30]. Thus Emx2 appears to act at two stages: to establish the domain where hem induction will occur, and later, to mediate the effects of hem signaling during further development of this region.

While these mechanisms serve to define the rostro-caudal extent of the hem, other mechanisms act to define the medio-lateral boundaries of the hem with the choroid plexus and the cortex. An early acting regulatory mechanism involving transcription factors of the *bHLH* family regulates the hem–choroid plexus boundary [31]. The hem and choroid plexus are defined by the differential expression of *Hes* and *Ngn* genes. At the time of boundary formation, *Hes* genes are enriched in the putative choroid plexus region, possibly as a result of the direct activation of these genes by *Bmps* from the roof plate. At the same time, this region downregulates *Ngn* gene expression, which continues to be maintained in the adjacent cortical hem. This downregulation of *Ngn* expression is important in establishing the choroid plexus fate and therefore delineating the hem–choroid plexus boundary [31].

## 4 Molecular mechanisms that act to position and specify the antihem

Three transcription factors, *Pax6*, *Tlx*, and *Gsh2*, are known to regulate the specification and positioning of the antihem. Its location at the PSB makes the antihem vulnerable to perturbations that disrupt dorsoventral patterning in the telencephalon.

The PSB is severely affected in the *Pax6* mutant. There is a ventralization of the pallial neuroepithelium of the *Pax6* mutant telencephalon, such that the VP and LP now express subpallial markers *Mash1*, *Gsh2* and *Dlx2* ([32,33,1,7,34,35]). *Tlx* mutants exhibit a similar, but less severe phenotype than *Pax6* mutants [36]. *Tlx* is expressed throughout the neuroepithelium, high at the lateral sulcus and on both sides of the PSB. As in the case of the *Pax6* mutant, the *Tlx* mutant too exhibits LGE characteristics at the expense of those of the VP [36]. In contrast, the *Gsh2* mutant displays the opposite phenotype, one in which pallial gene expression signatures are seen in subpallial domains such as the dLGE [7,35]. A detailed analysis of the interactions of *Pax6* and *Gsh2* reveals a cross-repressive mechanism, wherein Pax6 is required to induce VP-specific markers, and Gsh2 is necessary to suppress the expression of these genes in the dLGE, thereby restricting them to the VP [37].

## 5 “Organizer” functions

### 5.1 Hem

The cortical hem was considered to be analogous to the dorsal signaling center of the spinal cord, the roof plate, which also secretes Wnt and Bmp family molecules [12,38]. Bmp signaling from the roof plate is responsible for patterning adjacent neuronal fates [39], and ablation of the roof plate causes loss of specific neuronal populations [40]. A similar role for the cortical hem was proposed [12]. Supporting this hypothesis, the entire hippocampus is missing when the hem is deleted [41], or when a particular hem-specific signaling molecule, *Wnt3a*, is disrupted [42]. When components of the Wnt signaling cascade *Lef1* [43] or *Lrp6* [44] are disrupted, the dentate precursor pool is diminished and does not mature or migrate properly. But highly reduced cell populations of the dentate precursors were detected in each mutant [43,44]. Therefore, these studies were not able to separate a role for Wnt signaling in the expansion of the precursor population from one in which they act to specify of hippocampal cell fates [45].

**Fig. 3:**
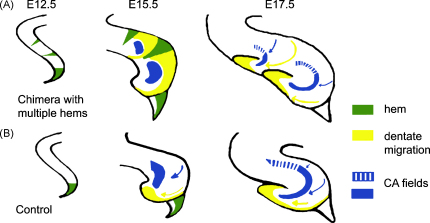
Ectopic hem induces and organizes multiple hippocampal fields. (A) and (B) are schematic representations of chimeric and control brains, respectively. The normal hem is seen at the medial extreme of the E12.5 telencephalic neuroepithelium. In the chimeras, Lhx2 mutant clusters form ectopic patches of hem in the medial telencephalon. By E15.5, control brains display markers for the hippocampal CA fields as well as for dentate granule cells. Both cell types originate in neuroepithelium and migrate away (blue and yellow arrows) to form the characteristic morphology of the Ammon's horn and the dentate gyrus by E17.5. In chimeric brains, CA and dentate cells are induced in appropriate spatial order adjacent to each ectopic hem, with the dentate granule cells immediately adjacent to the hem. By E17.5, the chimeras have assembled distinct dentate gyri and CA fields forming a double hippocampus. Modified from [17] [www.sciencemag.org]. (For interpretation of the references to color in this figure legend, the reader is referred to the web version of the article.)

The role of the cortical hem has also been tested in explant culture experiments in which the hem was either removed, or transplanted to ectopic locations of medial telencephalic preparations [46]. However, the age of the tissue used was E12.5, apparently too late for either perturbation to have any effect on hippocampal specification. Indeed, the authors concluded that the fine details of hippocampal field specification must have occurred by E12.5, even though overt differentiation of hippocampal fields occurs much later, from E15.5 [46]. Definitive evidence of the role of the cortical hem came from chimeras in which Lhx2 null cells, surrounded by wild-type cortical neuroepithelium, differentiated into ectopic hem tissue [17]. An ectopic hippocampus formed adjacent to each patch of hem, with spatially correct induction and positioning of multiple hippocampal fields (Fig. 3). This consolidated the cortical hem as an organizer for the hippocampus.

Which signaling molecules from the hem are critical for ectopic hippocampal induction? The literature strongly supports a role of Wnt signaling for this role. The *Wnt3a*, *Lef1*, and *Lrp6* mutant studies all indicate that Wnt signaling is necessary for hippocampal development [42–44]. Furthermore, ectopic activation of *Lef1* upregulated some hippocampal field markers in lateral neuroepithelium, demonstrating that Wnt signaling is sufficient for this process [47]. In contrast, Bmp signaling has not been implicated in hippocampal development. The *Bmpr1a* mutant, which lacks the telencephalic choroid plexus, appears to form a hippocampus [48]. In terms of regulating telencephalic neuronal development, Bmp signaling appears to act at earlier stages, including specifying the extreme medial fate of the choroid plexus [48–50]; causing cell death [11], and regulating the expression of *Lhx2* itself [21]. Furthermore, Bmp signaling is implicated in constraining the *Fgf8* domain, which in turn limits the domain of *Wnt* expression in the medial telencephalon [27]. Thus early actions of Bmp signaling may set in motion events which permit the formation of the cortical hem, which in turn induces the hippocampus. How signals from the hem bring about the specification of distinct hippocampal field identities remains an important open question.

An important issue is how the hem can direct not only the specification, but also the structural organization of multiple hippocampal fields. A clue comes from examining the radial glial palisade associated with the dentate migration. The organization of this palisade is thought to guide the dentate cells from their origin at the ventricular zone adjacent to the hem to their final location where they form the blades of the dentate gyrus [51]. Mangale et al. [17] report additional radial glial pallisades associated with ectopic hem tissue, which appear to guide distinct migratory streams terminating at each dentate gyrus (Fig. 3). The organization of the radial glial scaffolding itself is dependent on Wnt signaling [44]. Thus each patch of hem may be responsible for orienting the scaffolding adjacent to it, which would then guide the ectopically induced dentate cells to form an ectopic gyrus.

### 5.2 Antihem

In contrast to the organizer function of the hem, such a role for the antihem has yet to be identified. Nonetheless, the loss of the antihem is known to correlate with severe disruption of the radial glial pallisade at the PSB, raising a strong parallel with the role of the hem in organizing the hippocampal radial glia. *Tlx* mutants have fewer radial glial fibers at the PSB which do not appear to fasciculate to form a palisade [36]. *Pax6* is itself required for the differentiation of radial glia in the forebrain [52]. Not surprisingly, the radial glial palisade at the PSB is disrupted in the *Pax6* mutant [33,53,54]. Although markers identified the radial glial progenitor cell population, the fascicle itself could not be distinguished. Furthermore, interneurons produced within the subpallium were detected in greater numbers in the *Pax6* mutant cortex, suggesting that some feature of the normal PSB serves to restrict the tangential migration of interneurons [54]. Finally, *Pax6* mutant also displays profound defects in thalamocortical and corticofugal axon pathfinding. The underlying cause of this defect was suggested to be a combination of structural abnormalities and alterations in the expression of specific pathfinding molecules of the *Semaphorin* family at the *Pax6* mutant PSB [55].

Which other signaling molecules might mediate some of these defects? *Nrg1*, which is concentrated in the antihem, has been shown to be essential for the formation and maintenance of radial glial cells [56,57]. Drawing parallels with the hem, Wnt signaling might also regulate the radial glial pallisades organized by the antihem. *Wnt7b* is expressed adjacent to the antihem, in the dLGE [1]. The expression of the Wnt antagonist *Sfrp2* in the antihem may lead to a concentration of the Wnt signal to the subpallial side of the PSB [1], providing a positional signal to the radial glial pallisade.

Together, these studies support an integral role for the antihem in mediating axon guidance and cell migration, that of interneurons into the cortex as well as that of the derivatives of the lateral telencephalon, such as the olfactory cortex, the claustrum, and the amygdala. The latter role is likely to arise from the regulation of the radial glial pallisade at the PSB.

## 6 Derivatives: similarities and differences between the hem and the antihem

The hem was suggested to produce Cajal–Retzius cells [58] and this was demonstrated using genetic techniques to fate map the hem lineage [41,59]. The antihem also gives rise to Cajal–Retzius cells, as does the septum [9]. It is not at all clear why this earliest-born cell population has multiple origins. An attractive hypothesis proposes that this diversity of Cajal–Retzius cell progenitor zones may correlate with or regulate the development of cytoarchitectonic differences between the neocortex, olfactory cortex, and the hippocampus [9].

Reelin expression is a common feature to all these different types of Cajal–Retzius cells. Cajal–Retzius cells from the hem and antihem, but not those from the septum, express calretinin [9]. The hem lineage Cajal–Retzius cells express p73 [60], but those from the antihem and septum do not [9]. *Dbx1* expression, in contrast, marks cells from the antihem and the septum, but not those from the hem [9]. This unique combination of markers was used to selectively ablate specific sub populations, to examine possible functional roles arising from this diversity of Cajal–Retzius cell subtypes [9]. When antihem and septum derived Cajal–Retzius cells were ablated by expressing DTA (Diphtheria toxin) via the *Dbx1* locus, a significant loss of *reelin* expression was seen in the septum and pirifom cortex at E11.5, but this was compensated for by E14.5, presumably by Cajal–Retzius cells from other sources. However, there was a gross reduction in the thickness of the lateral cortex. This defect was selective for the lateral region, since the cingulate cortex appeared normal, indicating an important role for the antihem-derived Cajal–Retzius cells in regional cortical development [9].

A surprising, counterintuitive result came from experiments in which hem-derived Cajal–Retzius cells were ablated by expressing DTA via *Wnt3a* locus [41]. This caused a massive and near-complete depletion of Cajal–Retzius cells overlying the neocortex, which was apparently not rescued by migration of Cajal–Retzius cells from other sources. Despite this, neocortical lamination was unaffected [41]. Similarly, in *p73* mutants, which also display a loss of cortical Cajal–Retzius cells, neocortical lamination was normal except for the absence of the hippocampal fissure, which may be due to the loss of p73 itself [60]. Thus the precise role of hem-derived Cajal–Retzius cells continues to be elusive.

In addition to the production of Cajal–Retzius cells, the hem and the antihem also make unique contributions to the telencephalon. The hem produces the epithelial component of the choroid plexus, a secretory non-neuronal tissue that produces cerebrospinal fluid [61]. This is likely to be controlled by Bmp signaling [48] and by cross suppression between the *Ngn* and *Hes* genes [31]. The antihem is a major contributor of excitatory, pallial-derived cells of the amygdaloid complex. Gene expression studies [3,4,6] and genetic lineage tracing of the *Dbx1* lineage [62] indicates that the lateral and basomedial nuclei of the amygdaloid complex arise from the VP/antihem. The LP is thought to give rise to the basolateral nucleus which positions itself in between the two VP derived nuclei, and together these form the basolateral complex of the amygdala [4]. Mechanisms that disrupt the antihem also affect these structures, which are consequently greatly shrunken or missing in the *Pax6* [6] and the *Tlx* mutants [36]. In contrast, consistent with the antihem being spared in the *Lhx2* mutant, these structures are specified in the absence of Lhx2 [63]. The radial glial pallisade at the PSB is likely to participate in the migration of these cells to their final destinations, and indeed, such migrations have been visualized using GFP electroporation [64]. However, radial glia-independent type of “chain migration” has also been reported at the PSB [65]. Furthermore, migration of the basolateral complex of the amygdala does not seem to share mechanistic parallels with that in the cerebral cortex. Reelin is required for all neocortical cells to migrate to their appropriate destinations [66]. Cells of the superficial layers of the cortex require Cdk5 to migrate past the deep layers [67,68]. In contrast, cell migration of the basolateral amygdala is normal in the *Reelin* and the *Cdk5* mutants [2] suggesting that the mechanisms that regulate the assembly of the intricate structural complexity of the amygdaloid complex are far from simple, and as yet poorly understood.

## 7 Evolutionary perspectives

Both the hem and the antihem are evolutionarily ancient, having been identified in several vertebrate phyla. The hem has been identified in birds [69] and also in reptiles [70]. The antihem appears earlier, and was an important discovery as a ventral pallial territory in amphibians [71–73].

The positions of the hem and the antihem on the medial and lateral edges of the pallium, respectively, therefore preceded the expansion of the pallium in mammals. This motivates the speculation that the interactions of the hem and the antihem may have played a role in stimulating the expansion of the dorsal pallium. At the caudal telencephalon of the mouse embryo, where these two signaling centers almost meet (Fig. 1C), the intervening tissue produces an unusual stream of migrating cells, the caudal amygdaloid stream, that forms the nucleus of the lateral olfactory tract (layer 2; nLOT2). This is the only component of the amygdaloid complex that originates in the dorsal pallium, in contrast to other nuclei that arise from the lateral or ventral pallium [2]. The nLOT2 is also unique in its dependence on two modern mechanisms for cell migration: Cdk5 and Reelin. When either of these mechanisms are disrupted, the nLOT2 is selectively affected, whereas the rest of the amygdaloid complex is unperturbed [2]. Since the nLOT has itself been identified in reptiles [74], it too predates the appearance of laminated neocortex, derived from the mammalian dorsal pallium. The requirements of migration of the nLOT2 may in fact have presaged the Cdk5 and Reelin dependence of the mammalian neocortex. Whether the hem or the antihem regulate any aspect of the nLOT2 specification or migration is unknown, but their juxtaposition on either side of the nLOT2 primordium places them as potentially significant not only in the development, but also in the evolution of the cortex.
